# Machine Learning Applications for the Prediction of Bone Cement Leakage in Percutaneous Vertebroplasty

**DOI:** 10.3389/fpubh.2021.812023

**Published:** 2021-12-10

**Authors:** Wenle Li, Jiaming Wang, Wencai Liu, Chan Xu, Wanying Li, Kai Zhang, Shibin Su, Rong Li, Zhaohui Hu, Qiang Liu, Ruogu Lu, Chengliang Yin

**Affiliations:** ^1^Department of Orthopedics, Xianyang Central Hospital, Xianyang, China; ^2^Clinical Medical Research Center, Xianyang Central Hospital, Xianyang, China; ^3^Department of Orthopedics, The First Affiliated Hospital of Harbin Medical University, Harbin, China; ^4^Department of Orthopaedic Surgery, The First Affiliated Hospital of Nanchang University, Nanchang, China; ^5^Department of Dermatology, Xianyang Central Hospital, Xianyang, China; ^6^Xiamen Bank, Xiamen, China; ^7^The First Affiliated Hospital, Shaanxi University of Traditional Chinese Medicine, Xianyang, China; ^8^Department of Spine Surgery, Liuzhou People's Hospital, Liuzhou, China; ^9^Department of Electronic and Computer Science, University of Southampton, Southampton, United Kingdom; ^10^Faculty of Medicine, Macau University of Science and Technology, Macau, China

**Keywords:** percutaneous vertebroplasty, bone cement leakage, machine learning algorithms, prediction model, web calculator

## Abstract

**Background:** Bone cement leakage is a common complication of percutaneous vertebroplasty and it could be life-threatening to some extent. The aim of this study was to develop a machine learning model for predicting the risk of cement leakage in patients with osteoporotic vertebral compression fractures undergoing percutaneous vertebroplasty. Furthermore, we developed an online calculator for clinical application.

**Methods:** This was a retrospective study including 385 patients, who had osteoporotic vertebral compression fracture disease and underwent surgery at the Department of Spine Surgery, Liuzhou People's Hospital from June 2016 to June 2018. Combing the patient's clinical characteristics variables, we applied six machine learning (ML) algorithms to develop the predictive models, including logistic regression (LR), Gradient boosting machine (GBM), Extreme gradient boosting (XGB), Random Forest (RF), Decision Tree (DT) and Multilayer perceptron (MLP), which could predict the risk of bone cement leakage. We tested the results with ten-fold cross-validation, which calculated the Area Under Curve (AUC) of the six models and selected the model with the highest AUC as the excellent performing model to build the web calculator.

**Results:** The results showed that Injection volume of bone cement, Surgery time and Multiple vertebral fracture were all independent predictors of bone cement leakage by using multivariate logistic regression analysis in the 385 observation subjects. Furthermore, Heatmap revealed the relative proportions of the 15 clinical variables. In bone cement leakage prediction, the AUC of the six ML algorithms ranged from 0.633 to 0.898, while the RF model had an AUC of 0.898 and was used as the best performing ML Web calculator (https://share.streamlit.io/liuwencai0/pvp_leakage/main/pvp_leakage) was developed to estimate the risk of bone cement leakage that each patient undergoing vertebroplasty.

**Conclusion:** It achieved a good prediction for the occurrence of bone cement leakage with our ML model. The Web calculator concluded based on RF model can help orthopedist to make more individual and rational clinical strategies.

## Introduction

Percutaneous vertebroplasty (PVP) was a minimally invasive technique that uses liquid polymethylmethacrylate bone cement injected into the fractured vertebrae to reduce pain, strengthen the bone and prevent further compression of the vertebral body. PVP has become the mainstay treatment for osteoporotic and malignant vertebral fractures. Leakage of bone cement into the intervertebral disc increases the risk of adjacent vertebral fractures paraplegia due to extravasation of bone cement into the epidural or spinal cord compression associated with the intervertebral foramen (1). Besides, Leakage of bone cement into the paravertebral vein can lead to pulmonary embolism cardiac perforation, cerebral embolism and even death (2). Meanwhile, there are few reports of screening for independent risk factors and risk prediction models for bone cement leakage in patients undergoing orthopaedic percutaneous vertebroplasty.

Algorithmic decision support tools, computer-assisted navigation and surgical robots have served in the clinic (3, 4). Machine learning (ML) has been widely used in healthcare data analysis and some in spinal surgery (5, 6). By the comparison among ML algorithms, a better predictive model can be constructed to predict cement leakage after percutaneous vertebroplasty.

Some studies elaborated that age, bone mineral density (BMD), body mass index (BMI), volume of bone cement injections, and other underlying chronic diseases were risk factors for cement leakage (7). In addition, we added injury-to-operation time, hospitalization-to-operation time, and length of surgery.

Our study aimed to obtain the best ML algorithm, and constructed a big data risk prediction web calculator for postoperative cement leakage in patients undergoing percutaneous vertebroplasty. It ultimately provided a basis for future treatment and prevention strategies related to postoperative complications.

## Methods

### Study Population

All data for this study were available from the Department of Spine Surgery, Liuzhou People's Hospital. This study recorded the medical variables of patients hospitalized with or without cement leakage after undergoing percutaneous vertebroplasty, from June 2016 to June 2018. Retrospectively analyzed the basic patient's information, as well as baseline clinical characteristics, surgical details.

### Data Collection

To develop the target machine learning model, several variables were obtained, including patient age, height, weight, body mass index, bone mineral density, volume of bone cement injection, length of hospital stay to surgery, length of surgery, history of previous disease, and medication, as well as whether re-fracture occurred after surgery. Various machine learning algorithms such as logistic regression, random forest and other currently popular machine learning models were utilized in this study. Inclusion criteria for inpatient data were as follows: (1) osteoporotic vertebral fracture with moderate or severe low back pain which limited movement as the main complaint; (2) no significant improvement after active conservative treatment; (3) one or more vertebral fractures confirmed by imaging; (4) bone mineral density <2.5 by dual energy X-ray bone densitometry. In addition, exclusion criteria were: (1) fractures with symptoms of spinal nerve compression; (2) pathological vertebral fractures, such as spinal tumors, vertebral hemangiomas, spinal tuberculosis, etc.; and (3) patients with poor physical fitness and intolerance to surgical treatment. This study was approved by the Institutional Review Board of the Liuzhou People's Hospital before data collection and analysis, the IRB approval number is 2020 (KY-E-22-01).

### Surgical Strategy

Patients undergo individualized PVP in the prone position with local anesthesia. All PVPs were performed with a unilateral lateral approach through the vertebral arch. The puncture was performed through the vertebral arch under fluoroscopic guidance. The puncture point is located at the intersection of a cephalolateral with a horizontal line 5–15 mm from the arch projection and a lateral vertical line ~ 30 mm from the posterior midline. Under fluoroscopy, a 3.5 mm needle was moved along the root of the arch, the head adjustment and abduction angle of the needle bundle was allowed until the junction of the anterior third of the vertebral body was reached. The spread and distribution of the bone cement was monitored under fluoroscopy while the patient was tested for bilateral lower limb movement. After that, needle was withdrawn, covered with a sterile dressing and secured with pressure. Pre- and post-operative imaging is routinely performed in screened cases to determine the degree of compression of the fractured vertebral body, the height of the vertebral body, the integrity of the posterior wall of the vertebral body, the amount of cement injected intraoperatively, whether the cement leaks, and whether the vertebral body is re-fractured postoperatively.

### Statistical Analyses

Exploring the independent risk factors for PVP, multivariate logistic regression analysis was applied to calculate the odds ratio (OR) with 95% confidence intervals (CI) using a backward stepwise selection method. The value of OR >1 indicates that the variable is a risk factor. *P* < 0.05 was regarded as statistically significant. The predictive ability of ML algorithms has outperformed traditional regression methods in large databases (8). We used six different ML algorithms to analyze our data: logistic regression (LR), Gradient boosting machine (GBM), Extreme gradient boosting (XGB) Random Forest (RF), Decision Tree (DT) and Multilayer perceptron (MLP). After training, the one with the highest average AUC was chosen as the best algorithm. Furthermore, the ML-based model was tuned to avoid overfitting and the accuracy of the algorithm was tested using ten-fold cross-validation method.

In our study, the components and proportions of multiple relevant factors related to PVP were analyzed and visualized by heat map for the collected patient data. Furthermore, the heat map can also be used to identify data of outrageous quality during the actual analysis and assist in quality control.

We first used a multiple regression model as a benchmark for data evaluation and ML (which can handle a large number of input variables, assess the importance of variables when deciding on categories, and detect variable interactions). In the performance comparison of ML algorithms, the more AUC close to one, the better performance these classification model obtained. Based on the best execution model, a web calculator was created in this paper which implemented the prediction of illness for inputting patient data. The calculator allowed the surgeon to assess the risk of possible postoperative cement leakage in patients undergoing percutaneous vertebroplasty.

## Results

### Demographics Features

The clinical history of 385 patients who underwent PVP was summarized in this study, the number of patients who developed post-operative cement leakage was 81 and the number of patients who did not develop cement leakage was 304 (Table 1). Among these observed subjects, the median age was 75.4 years (IQR:68.3, 80.7), the median height of these patients was 155 cm (IQR:149,161), the median weight of the patients was 47kg (IQR:40,58). The median BMD was 4.4 (IQR:3.9,5.0). The *P* values of these indicators were 0.987, 0.2082, 0.948, and 0.1833, respectively. None of them were statistically different. It worth noting that the 205 patients (53.25%) who did not have multiple vertebral fractures (*P* < 0.05). Besides, no cement leakage occurred in 177 patients (58.22%) and cement leakage occurred in 28 patients (34.57%).

**Table 1:** Baseline table of patients with and without cement leakage.

**Variables**	**level**	**Overall**	**No**	**Yes**	***p*-value**
		**(*N* = 385)**	**(*N* = 304)**	**(*N* = 81)**	
Age (median [IQR])	NA	75.400 [68.300, 80.700]	75.650 [68.300, 80.750]	74.900 [68.200, 80.600]	**0.987**
High (median [IQR])	NA	155.000 [149.000, 161.000]	155.000 [148.000, 160.000]	155.000 [149.000, 162.000]	**0.2082**
Weigh (median [IQR])	NA	47.000 [40.000, 58.000]	47.000 [41.000, 58.000]	47.000 [40.000, 60.000]	**0.948**
BMI (median [IQR])	NA	19.899 [17.116, 23.873]	19.905 [17.360, 23.905]	19.819 [16.437, 23.613]	**0.4335**
BMD (median [IQR])	NA	4.400 [3.900, 5.000]	4.400 [3.900, 5.000]	4.600 [4.100, 4.900]	**0.1833**
Injection volume of bone cement (median [IQR])	NA	4.000 [3.500, 5.000]	4.000 [3.500, 5.000]	4.000 [4.000, 5.000]	**0.0558**
Hospital stay to surgery (median [IQR])	NA	5.000 [4.000, 6.000]	5.000 [3.000, 6.000]	5.000 [4.000, 7.000]	**0.1086**
Injury to surgery (median [IQR])	NA	14.000 [8.000, 30.000]	14.000 [8.000, 30.000]	16.000 [10.000, 33.000]	**0.2193**
Antiosteoporosis (%)	No	245 (63.64)	192 (63.16)	53 (65.43)	**0.804**
	Yes	140 (36.36)	112 (36.84)	28 (34.57)	
Multiple (%)	No	205 (53.25)	177 (58.22)	28 (34.57)	**0.0002**
	Yes	180 (46.75)	127 (41.78)	53 (65.43)	
Steroid (%)	No	320 (83.12)	248 (81.58)	72 (88.89)	**0.1634**
	Yes	65 (16.88)	56 (18.42)	9 (11.11)	
Re-fracture (%)	No	327 (84.94)	260 (85.53)	67 (82.72)	**0.6502**
	Yes	58 (15.06)	44 (14.47)	14 (17.28)	

**Figure 1:**
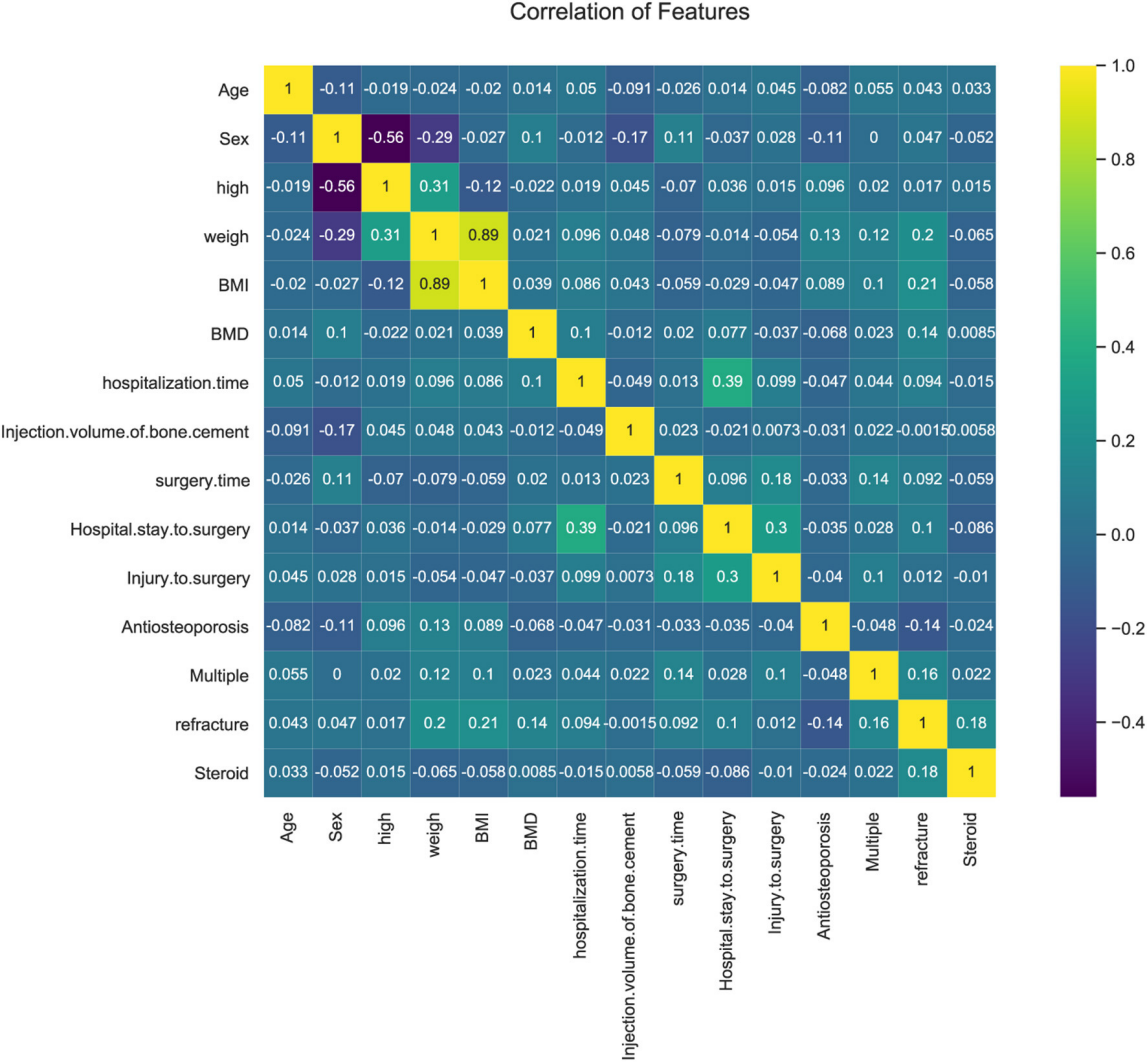
Heat map of the correlation of patient's clinical features.

In the correlation heat map (Figure 1), 15 highly correlated features were chosen as predictors. Weight was absolutely correlated with BMI and to some extent with height. As BMI got a better indicator of obesity, we only considered BMI rather than height or weight.

### Univariate and Multivariate Logistic Regression Analyses of Cement Leakage

In terms of univariate analyzing (Table 2), age, height, weight, body mass index, bone mineral density, volume of bone cement injected, length of hospital stay, and intraoperative injury didn't have significant difference in occurrence of bone cement leakage in the overall population (*p* > 0.05).

**Table 2:** Univariate and multivariate logistic regression of bone cement leakage.

**Variables**	**Univariate OR (95% CI)**	***P-*value**	**Multivariate OR (95% CI)**	***p*-value**
Age (years)	1.002 (0.976–1.029)	**0.868**	/	**/**
Sex				
Female	Ref	**Ref**	/	**/**
Male	0.799 (0.421–1.514)	**0.492**	/	**/**
BMI	0.992 (0.946–1.040)	**0.757**	/	**/**
BMD	1.272 (0.878–1.844)	**0.202**	/	**/**
Hospitalization time (days)	1.021 (0.973–1.072)	**0.393**	/	**/**
Injection.volume.of.bone.cement (ml)	1.280 (1.0042–1.633)	**0.046**	1.283 (1.004-1.639)	**0.046**
Refracture				
No	Ref	**Ref**	/	**/**
Yes	1.234 (0.639–2.385)	**0.530**	/	**/**
Surgery time (min)	1.016 (1.004–1.028)	**0.005**	1.014 (1.002-1.027)	**0.017**
Hospital.stay.to.surgery (days)	1.058 (0.983–1.140)	**0.130**	/	**/**
Injury to surgery (days)	0.998 (0.992–1.004)	**0.711**	/	**/**
Anti-osteoporosis therapy				
No	Ref	**Ref**	Ref	**Ref**
Yes	0.905 (0.541–1.513)	**0.705**	/	**/**
Multiple vertebral fracture				
No	Ref	**Ref**	Ref	**Ref**
Yes	2.638 (1.581–4.399)	**0.000**	2.456 (1.460–4.129)	**0.000**
Steroid use				
No	Ref	**Ref**	Ref	**Ref**
Yes	0.553 (0.261–1.173)	**0.122**	/	/

In the multivariate logistic regression analysis, all parameters from the univariate analysis above were included. OR value showed the relative risk of bone cement leakage. The results showed that age (OR 1.002, 95% confidence interval 0.976–1.029), male (OR 0.799, 95% confidence interval 0.421–1.514), body mass index (OR 0.992, 95% confidence interval 0.946–1.040), bone mineral density (OR 1.272, 95% confidence interval 0.878–1.844), and length of hospital stay (OR 1.021, 95% confidence interval 0.973–1.072). Injection volume of bone cement, Surgery time, and Multiple vertebral fracture were all independent predictors for bone cement leakage. Besides, Multiple vertebral fractures (OR 2.638, 95% confidence interval 1.581–4.399), indicating a statistical difference (*P* < 0.05).

### Performance of Machine Learning Algorithms

**Figure 2:**
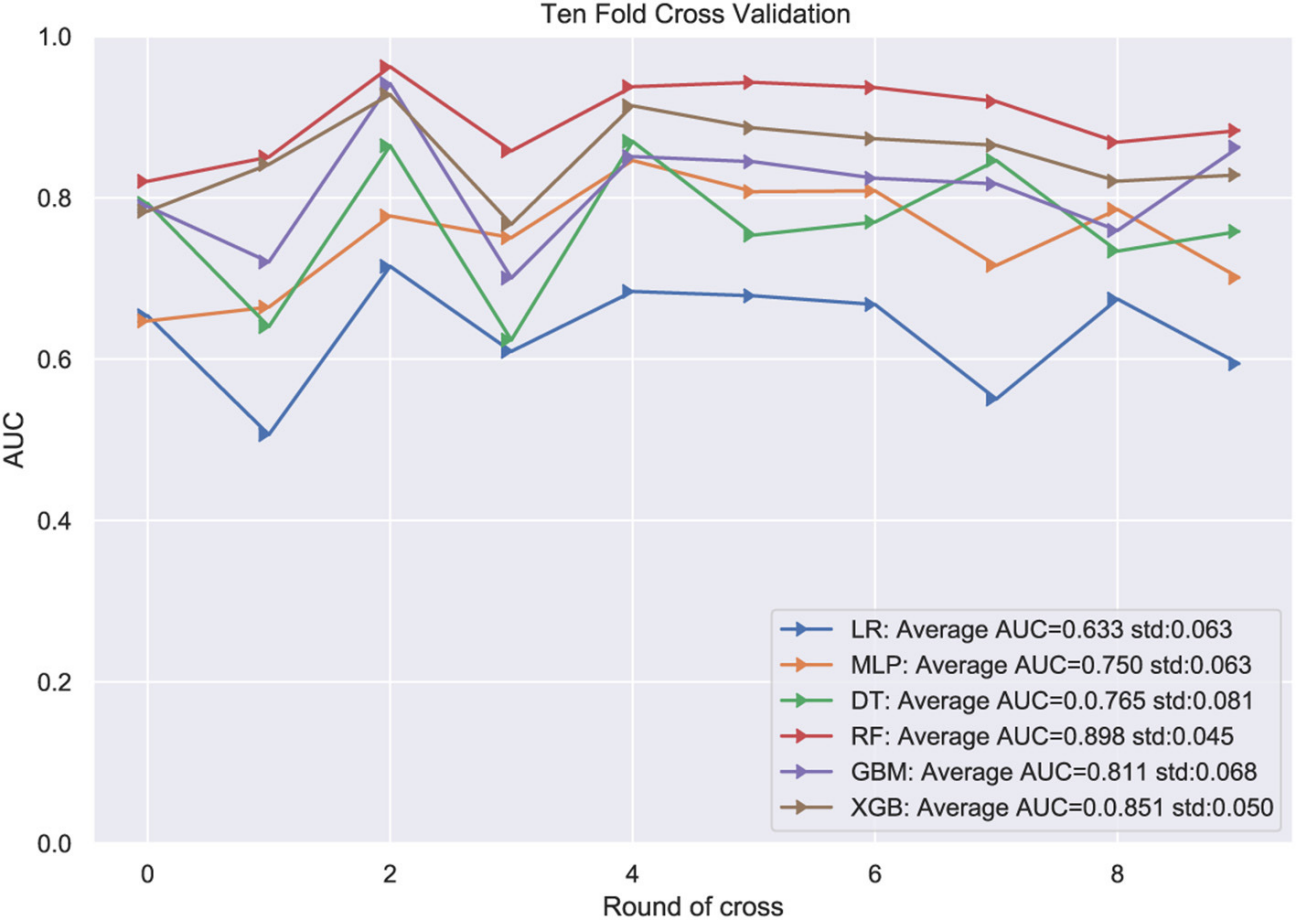
10-fold cross validation test. LR, logistic regression; MLP, Multilayer perceptron; DT, Decision Tree; RF, Random Forest; GBM, gradient boosting machine; XGB, extreme gradient boosting.

To compare the predictive performance of these six different ML algorithm models, 10-fold cross validation was applied in this study (Figure 2). As shown in the figure, the random forest model performed best in predicting bone cement leakage with an average AUC of 0.898. The remaining models were sorted in descending order depending on prediction performance. Therefore, we finally regarded the RF model as the preferred prediction model.

### Relative Importance of Variables in Machine Learning Algorithms

**Figure 3:**
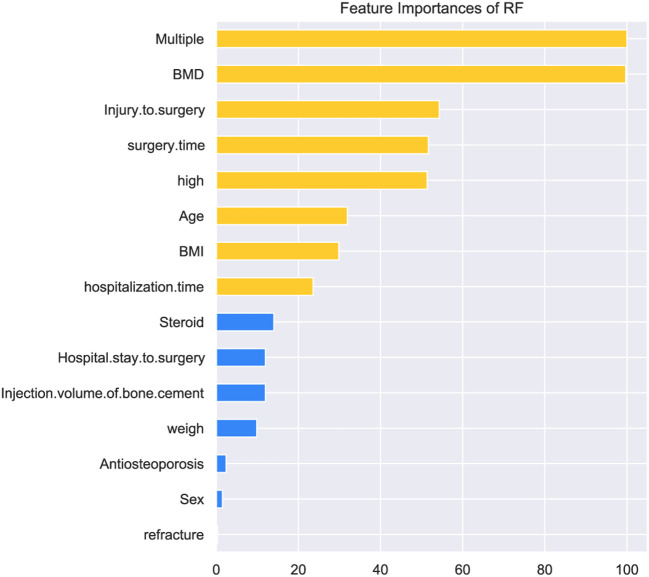
Patient clinical feature importance of Random Forest.

The feature importance were different from each other while multiple vertebral fractures and bone density were at first. Intraoperative injury, length of surgery, height, age, body mass index and length of hospital stay were arranged in order of importance. Then, variables such as steroid hormone use, length of hospital stay to surgery, volume of bone cement injection, body weight, anti-osteoporosis medication, and gender had bad performance in predicting the contribution of bone cement leakage and were ranked in descending order (Figure 3). Particularly, re-fracture had no effect on bone cement leakage.

### Web-Based Calculator

**Figure 4:**
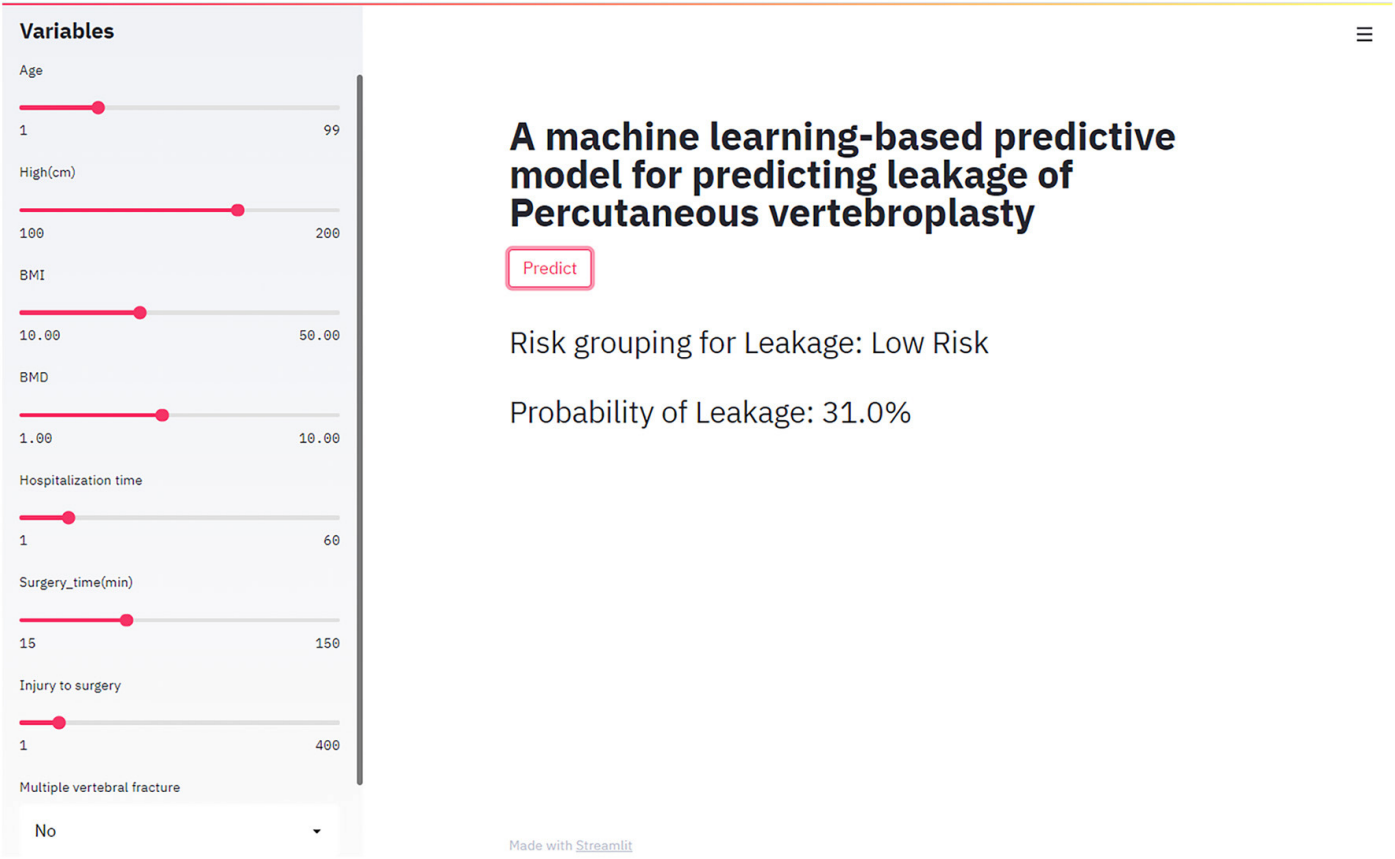
The web-based calculator for predicting bone cement leakage in patients with percutaneous vertebroplasty.

In this study, a web-based online calculator based on random forest model was developed. By entering variables that characterize the clinical profile of patients about to undergo percutaneous vertebroplasty, clinicians were able to predict the risk of cement leakage of their patients (https://share.streamlit.io/liuwencai0/pvp_leakage/main/pvp_leakage) (Figure 4).

## Discussion

Our research work used clinical data from patients to produce a risk calculator for bone cement leakage using ML algorithm, which using multiple variables to predict the risk oucomes of patients. The model was established based on a range of predictions and tested for accuracy and reasonableness through 10-fold cross validation. This artificial intelligence-based strategy can be exploited by clinicians to help them select more rational treatment responses (9).

ML contributes to the paradigm shift inherent in healthcare, where computers learn from patient data without being explicitly programmed to do the task (10, 11). It has the advantage of being highly capable, objective and repeatable in processing large data sets, reliable data (12, 13). It also has the potential to improve the quality of early diagnosis, identify disease progression and can increase the likelihood of predicting specific patient outcomes in orthopaedics, such as outcome scores, risk of complications and survival of implants (14). These benefits can facilitate the sharing of decision-making information between clinicians and patients and promote the effective planning and rational use of healthcare services (15, 16). In addition, the model can be retrained over time in order to improve predictive accuracy (17).

Percutaneous cement augmentation has become a common treatment for osteoporotic fractures of the thoracolumbar spine. Bone cement filling is an essential component that reconstructs the strength and biomechanics of the fractured vertebra and prevents re-fracture (18, 19). The incidence of bone cement leakage ranges from 5 to 87% (20, 21) and pulmonary embolism or spinal injury due to bone cement leakage is rarely reported (22, 23). Bone mineral density was an important indicator of bone strength and was the most effective predictor of fracture risk (24). Guo et al., found that the volume of injected bone cement was an independent risk factor for cement leakage (25), so we added this variable to our model algorithm.

In this study, we compared multiple machine learning algorithms for predicting the risk of bone cement leakage in patients undergoing percutaneous vertebroplasty. The comparison of ML algorithms revealed that the random forest performed best. To better utilize the model, we further built a web calculator estimating the probability of bone cement leakage in patients.

It was important to note that preoperative variables including age, body mass index and bone mineral density were significant predictors that can be used to assess the risk of cement leakage and thus help clinicians to choose the best treatment and know the prognosis of the patient. However, these factors in previous studies were based on preoperative information and were deficient to achieve reliable predictions. In addition, few studies have assessed the predictive value of intraoperative factors. Therefore, we established the key variable of intraoperative cement injection volume, which not only ensures that correct intraoperative manipulation and restoration of vertebral strength can be safely performed, but also helps us to predict the extent of cement leakage.

This study aimed to use a dataset consisting of 385 patients to help us select the best machine learning prediction. Our work had several advantages. First, few studies have used machine learning methods to assess the risk of bone cement leakage during percutaneous vertebroplasty. Besides there were few studies in the existing literature that have applied ML algorithms to orthopaedic surgical complications. As far as we know, this study was the first to use ML algorithms to develop a web calculator that allows orthopaedic surgeons to use clinical data for real-time risk assessment of surgical complications. Last but not least, by comparison, our model shows superior predictive power and based on this we have built an online risk assessment program that allows orthopaedic surgeons to calculate risk variables and provide individualized surgical treatment to patients.

However, there are some limitations in this study. Firstly, the nature of retrospective studies can lead to subjective bias and selection bias. Secondly, ML algorithm model we developed was limited to one hospital. This may limit its use in other regions, which requires more use for further validation. Thirdly, the predictive performance was not good enough.

In machine learning, supervised learning identified the relationship between input data (e.g., patient clinical characteristics) and output data (e.g., bone cement leakage), which can help us predict outcomes. This has done through regression or classification. In classification tasks, the analysis was usually implemented by using logistic regression or decision trees. There were several studies on the latter shown that patient's age, associated comorbidities, functional status, etc. could lead the patient not being discharged as planned (26, 27). Furthermore, as more data being obtained, models can be retrained over time to improve prediction accuracy. Of course, clinicians always have the final decision when it comes to interpretation based on their domain expertise.

In conclusion, in this article we found that the random forest algorithm had good performance while applied as a tool for predicting bone cement leakage after orthopaedic surgery, Meanwhile, it was reasonably accurate and simple to use. Using this web-based calculator we could effectively prevent complications after percutaneous vertebroplasty and assist doctors in their surgical decisions. It is hoped that in the near future this web calculator will be able to cover a wide range of clinical variables, even at the molecular and cellular level, so that it can be used more accurately to assist a wider population.

## Data Availability Statement

The raw data supporting the conclusions of this article will be made available by the authors, without undue reservation.

## Ethics Statement

This study data was approved by the Institutional Review Board of the Liuzhou People's Hospital before data collection and analysis, the IRB approval number is 2020 (KY-E-22-01).

## Author Contributions

CY and RL: designed the study. WLL, JW, and WLiu: performed the study and analyzed the data. WLL and JW: wrote the manuscript. QL and ZH: provided the expert consultations and clinical suggestions. CX and WYL: conceived of the study, participated in its design, and coordination. KZ, SS, and RL: helped to draft the manuscript. All authors contributed to the article and approved the submitted version.

## Conflict of Interest

The authors declare that the research was conducted in the absence of any commercial or financial relationships that could be construed as a potential conflict of interest.

## Publisher's Note

All claims expressed in this article are solely those of the authors and do not necessarily represent those of their affiliated organizations, or those of the publisher, the editors and the reviewers. Any product that may be evaluated in this article, or claim that may be made by its manufacturer, is not guaranteed or endorsed by the publisher.

## Abbreviations

ML: machine learning
LR: logistic regression
GBM: Gradient boosting machine
XGB: Extreme gradient boosting
RF: Random Forest
DT: Decision Tree
MLP: Multilayer perceptron
AUC: Area Under Curve
PVP: Percutaneous vertebroplasty
BMD: bone mineral density
BMI: body mass index
OR: odds ratio
CI: confidence intervals.
